# The correlations between dimensions of the normal tendon and tendinopathy changed Achilles tendon in routine magnetic resonance imaging

**DOI:** 10.1038/s41598-021-85604-9

**Published:** 2021-03-17

**Authors:** Pawel Szaro, Khaldun Ghali Gataa

**Affiliations:** 1 Department of Radiology, Institute of Clinical Sciences, Sahlgrenska Academy, University of Gothenburg, Göteborgsvägen 31, 431 80 Gothenburg, Sweden; 2 Department of Musculoskeletal Radiology, Sahlgrenska University Hospital, Gothenburg, Sweden; 3 Department of Descriptive and Clinical Anatomy, Medical University of Warsaw, Warsaw, Poland

**Keywords:** Muscle, Tendons

## Abstract

This comparative study aimed to investigate how tendinopathy-related lesions change correlations in the dimensions of the Achilles tendon. Our experimental group included 74 patients. The mean age was 52.9 ± 10.4 years. The control group included 81 patients with a mean age was 35.2 ± 13.6 years, *p* < .001. The most significant difference in correlation was the thickness of the tendon and the midportion's width, which was more significant in the tendinopathy (r = .49 vs. r = .01, *p* < .001). The correlation was positive between width and length of the insertion but negative in normal tendons (r = .21 vs. r = − .23,* p* < .001). The correlation was between the midportions width in tendinopathy and the tendon's length but negative in the normal tendon (r = .16 vs. r = − .23, *p* < .001). The average thickness of the midportion in tendinopathy was 11.2 ± 3.3 mm, and 4.9 ± 0.5 mm in the control group, *p* < .001. The average width of the midportion and insertion was more extensive in the experimental group, 17.2 ± 3.1 mm vs. 14.7 ± 1.8 mm for the midportion and 31.0 ± 3.9 mm vs. 25.7 ± 3.0 mm for insertion, respectively, *p* < .001. The tendon's average length was longer in tendinopathy (83.5 ± 19.3 mm vs. 61.5 ± 14.4 mm, *p* < .001). The dimensions correlations in normal Achilles tendon and tendinopathic tendon differ significantly.

## Introduction

The spiral arrangement of the Achilles tendon is well-known^1–3^. The twisted structure of the Achilles tendon is most prominent in the midportion and is likely to play a role in forming the hypovascular zone^1,4^. Decreased vascularization may result in delayed healing and greater susceptibility to injury. Achilles tendinopathy is a common problem, affecting athletes more than the general population^5,6^. Diagnosis of tendinopathy is often clinical; however, diagnostic imaging plays an important role^7^. Alterations in the Achilles tendon's dimensions are frequently a sign of injury or degeneration^8^.

Because of better availability and lower cost, ultrasound is applied more often than MRI. The indisputable advantage of MRI is independence from the operator. Some studies suggest that ultrasound and MRI efficiency in the diagnosis of tendinopathy is similar^9,10^. However, there are no large population studies on the same group comparing ultrasound and MRI effectiveness in diagnosing Achilles tendinopathy.

It is estimated that approximately half of athletes may have Achilles tendinopathy^5,6^. In asymptomatic athletes, Achilles tendon abnormalities are relatively common^11^. Diagnostic imaging of early tendinopathy may be challenging because of scarce clinical and radiological signs. Diagnostic imaging in sports medicine aims to detect subclinical tendinopathy changes, which allows for early prevention of Achilles tendon rupture; therefore, appropriate patient selection is critical.

Diagnostic problems related to tendinopathy at an early-stage result from a short T2-time result in no signal or a low signal with minor pathological changes within the Achilles tendon. The use of novel MRI techniques such as T2-mapping, modification of classical diffusion tensor imaging (DTI) MRI, magnetic resonance spectroscopy, or sodium MRI, mainly used in scientific research, gives hope for increased MRI effectiveness detecting subclinical changes in the Achilles tendon in the future. At the present stage, clinical examination, and in doubtful cases, ultrasound or MRI are methods used to diagnose pain and Achilles tendon dysfunction. MRI findings regarding a painful Achilles tendon may be diverse. The most common causes of painful Achilles tendon are overuse and midportion tendinopathy. In addition to the two causes of Achilles tendon pain, there are also slightly rarer ones, such as paratendonitis, partial ruptures, enthesitis in arthritis, bursitis, or tumors. MRI plays an important role in excluding these pathologies.

In some patients, an early sign of Achilles tendinopathy is tendon thickening. Often, changes in the Achilles tendon signal are not yet observed at this stage. There is a lack of MRI studies on large material regarding changes in the dimensions of the Achilles tendon in patients with tendinopathy was performed. Thus, this study aimed to investigate how tendinopathy-related lesions change correlations in the Achilles tendon's dimensions. We hypothesize that correlations between measurements of the normal and tendinopathy changed Achilles tendon differ.

## Material and methods

We rereviewed MRIs of the Achilles tendon and the ankle of 74 patients with clinically and radiologically proven Achilles tendinopathy, and 81 patients with a normal Achilles tendon. All examinations were clinically indicated. Our experimental group included 28 females and 46 males. The age range was 29–72 years, with a mean of 52.9 ± 10.4 years. The right ankle was examined in 32 cases, the left one in 42 cases. The control group included 33 females and 48 males, *p* = .32. The age range was 18–68 years, with a mean of 35.2 ± 13.6 years, *p* < .001. The right ankle was examined in 47 cases, the left one in 34 cases, *p* < .05.

Inclusion criteria. The experimental group consisted of patients whose MRI examinations were performed from January 2018 to April 2020 due to Achilles tendon pain. The control group included consecutive normal MRI examinations performed at the same period as the experimental group for non-traumatic indications.

The MRI protocol may vary between cases because imaging was performed on different MRI machines; the most come from the 3 T machine. All examinations were performed with a dedicated ankle coil and the availability of at least the following sequences: PD or T2 in the sagittal plane (at least one with fat suppression) and T1. In most cases, the following protocol was used: PD TSE (turbo spin-echo) in the sagittal plane: TE (echo time) 30 ms, TR (repetition time) 2000–5000 ms. T2 TSE SPAIR (spectral attenuated inversion recovery) in the sagittal plane: TE 60 ms, TR 3000–5000 ms, T1 SE (spin echo) in transverse plane: TE 10 ms, TR 450–750 ms, T1 TSE in the sagittal plane: TE 11.5 ms, TR 450–470 ms. Slice thickness was 2–3 mm. A dedicated coil was used. Tendinopathy was considered to be present on MRI if the Achilles tendon was more or 6 mm^12^, the anterior outline of the tendon was convex, the fibril structure of the tendon was blurred, and the signal of the tendon was abnormal. Clinical suspicion of midportion tendinopathy was included in the referral letter.

Exclusion criteria were the Achilles tendon rupture (18 cases excluded), insertional tendinopathy (13 cases excluded), presence of orthopedic hardware due to possible artifacts (5 cases excluded), a history of the previous fracture of the calcaneus or talus (3 cases excluded), and obvious abnormality in relation to the Achilles tendon (2 cases excluded), motion artifacts (2 cases excluded), clinical suspicion of spondylarthritis (1 case excluded). In total, 72 patients in the experimental and 81 in the control group fulfilled the inclusion and exclusion criteria. All examinations were reviewed twice, and the final results were made by consensus.

**Figure 1:**
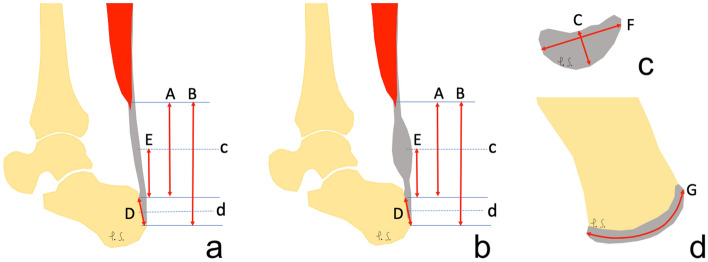
Dimensions of the Achilles tendon. (**a**) and (**b**) sagittal section, (**c**) and (**d**) transverse section. Section c was made at level E. Section d was made in the middle of distance D. A, B, C, D, E and F – dimensions of the Achilles tendon, as explained in the Material and Methods.

Protocol and definition of the measurements included in our study. The shortest Achilles tendon length was defined as the distance between the lowest outline of the soleus muscle belly to the superior outline of the Achilles insertion on the sagittal section (distance A), Fig. 1. The localization of the lowest point of the soleus was determined on the transverse section, then this point was identified on the sagittal section, and finally and the distance A was assessed. Subsequently, the longest Achilles tendon length was defined as the distance between the lowest outline of the soleus muscle belly to the lower contour of the Achilles tendon insertion (distance B), Fig. 1. Distance B was assessed on the sagittal section. The anterior–posterior dimension of the thickest part of the Achilles tendon was assessed on the axial plane (distance C), Fig. 1. The Achilles tendon insertion length was defined as the distance from the point where the edge of the calcaneus cortex was distinguishable from the anterior outline of the Achilles tendon to the most distal part of the bony attachment (distance D), Fig. 1. Dimension E is the distance from the upper outline of the insertion to the level at which dimension C was measured, Fig. 1. The width of the midportion (distance F) was assessed at the same level as the distance C on the axial section. The Achilles tendons insertion width (distance G) was assessed in the middle of the D dimension on the axial section, Fig. 1.

### Statistical analysis

The mean, standard deviation, minimum, and maximum of all tendon dimensions were estimated. To minimize measurement errors, all measurements were performed twice; subsequently, the arithmetic mean value was calculated. Repeated measures (test–retest) reliability analyses utilized interclass correlation coefficients (ICC), and 95% confidence intervals were obtained. We interpreted ICC values according to Fleiss et al.^13^, where a value of 0.75 or greater indicates excellent reliability; 0.40 to 0.75, fair to good reliability; and 0.40 or less poor reliability^14^. We calculated the standard error of measurement (SEM)^15^. The Shapiro–Wilk W test was used to verify the distribution of variables, and, once a normal distribution was confirmed, the t-test was used. Statistical significance was set at α = 0.05. Statistical correlations between the continuous data were assessed using the Pearson correlation coefficient.

The Swedish Ethical Review Authority approved the study and waived the need for informed consent (2020-06177). Our study was performed in accordance with relevant named guidelines and regulations.

## Results

**Figure 2:**
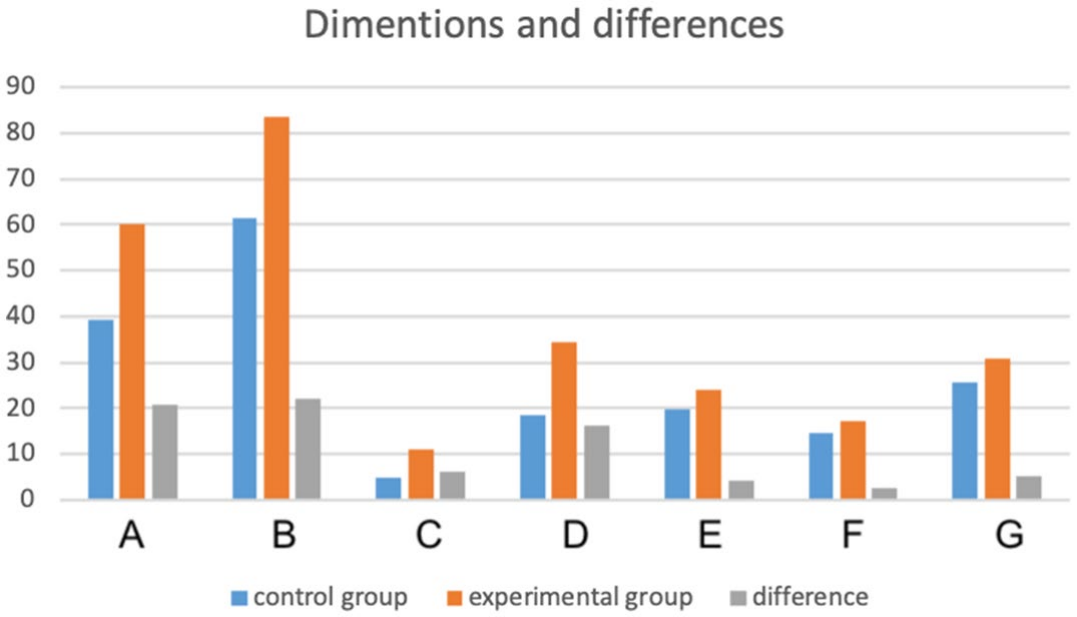
Differences in the average dimensions of the Achilles tendon in experimental and control group. The vertical axis represents millimeters, horizontal axis represents dimensions A–G; definitions are in the Material and Methods. The figure was prepared in Excel (version 16.46).

**Figure 3:**
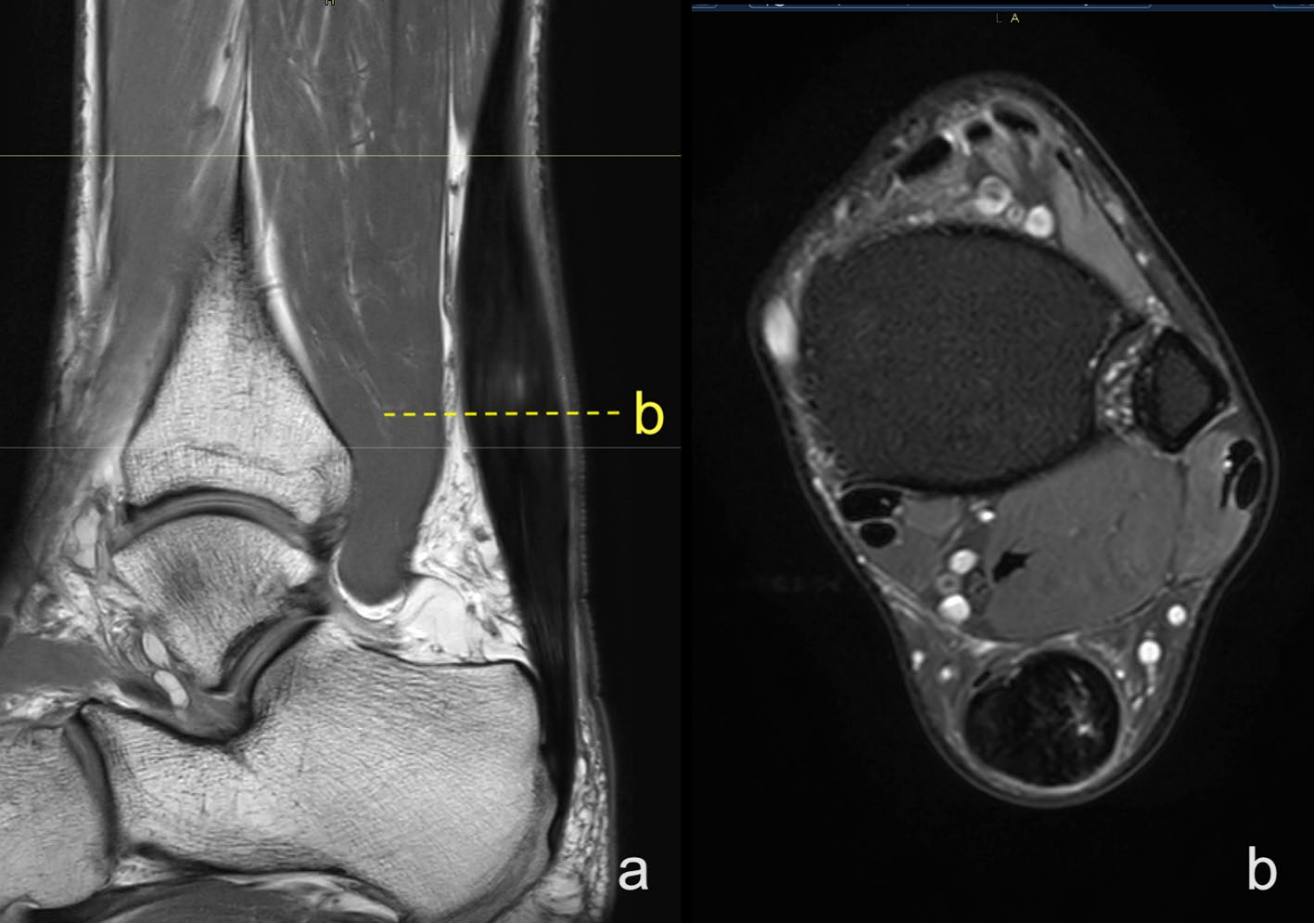
Midportion tendinopathy of the right Achilles tendon in a 45-year-old male who used to run about 3–5 km per day presenting with Achilles pain. Clinical diagnosis of tendinopathy was made with a suspicion of partial rupture. MRI showed a spindle-shaped Achilles tendon with alterations in the signal in the thickened part. (**a**)- Sagittal section, PD- weighted TSE, (**b**) axial section PD- weighted SPAIR.

**Figure 4:**
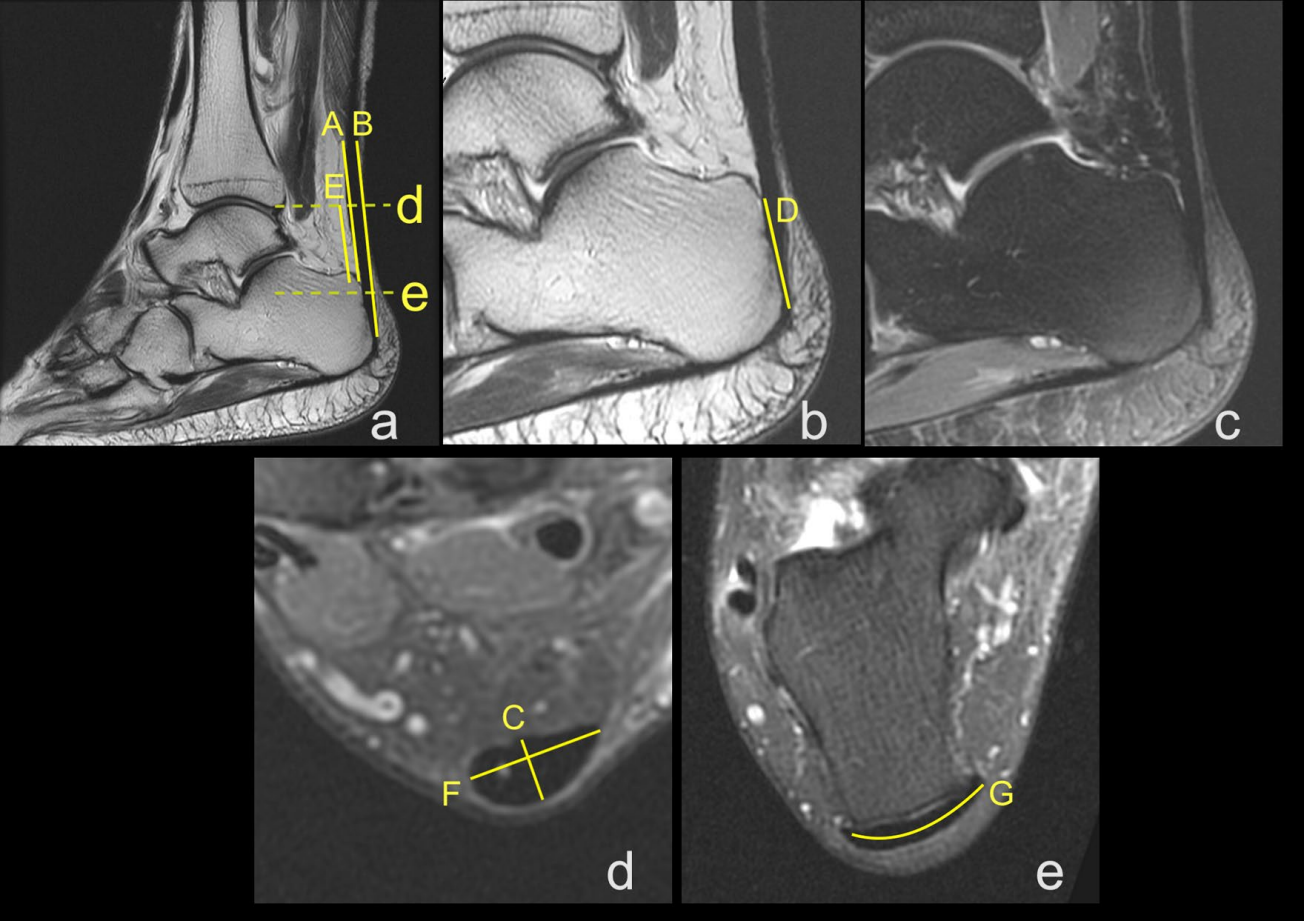
Insertion of the left Achilles tendon. A 61-year-old male who suffered from Achilles pain for eight months, with acute Achilles tendon pain one week before examination. Tendinopathy with partial rupture was suspected. A, B, C, D, F, G—dimensions of the Achilles tendon; definitions in the Material and Methods. (**a**) and (**b**) Sagittal T2-weighted FSE (fast spin echo), (**c**)- sagittal PD-weighted FS (fat suppression), (**d**) and (**e**) axial PD-weighted FS.

**Figure 5:**
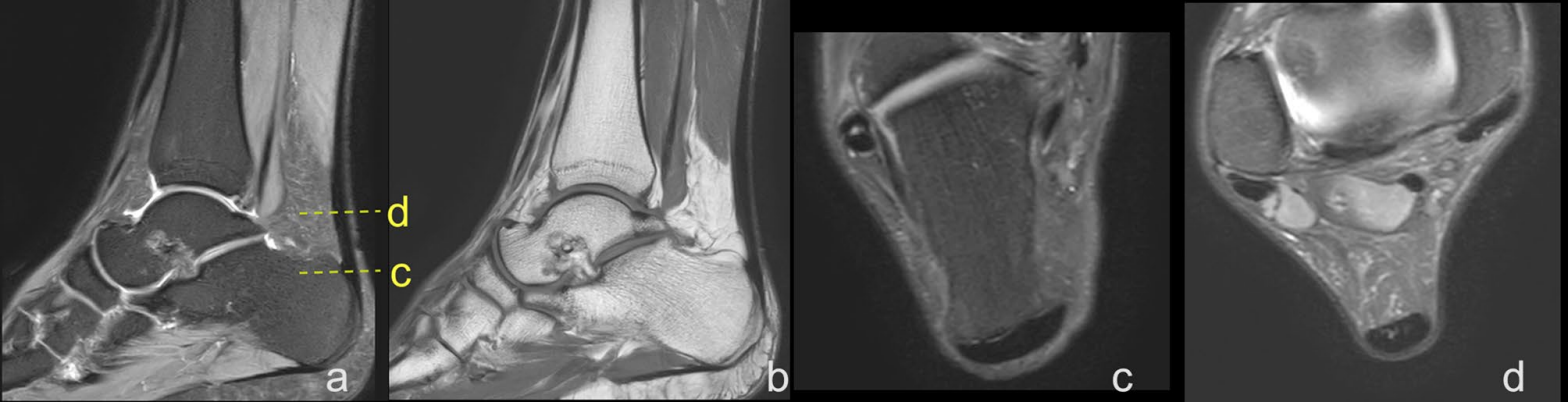
The normal Achilles tendon. A 34-year-old female with clinical suspicion of a nerve tumor on n. tibialis. (**a**) PD- weighted SPAIR, (**b**) T1-weighted, (**c**) and (**d**) PD- weighted TSE SPAIR.

**Figure 6:**
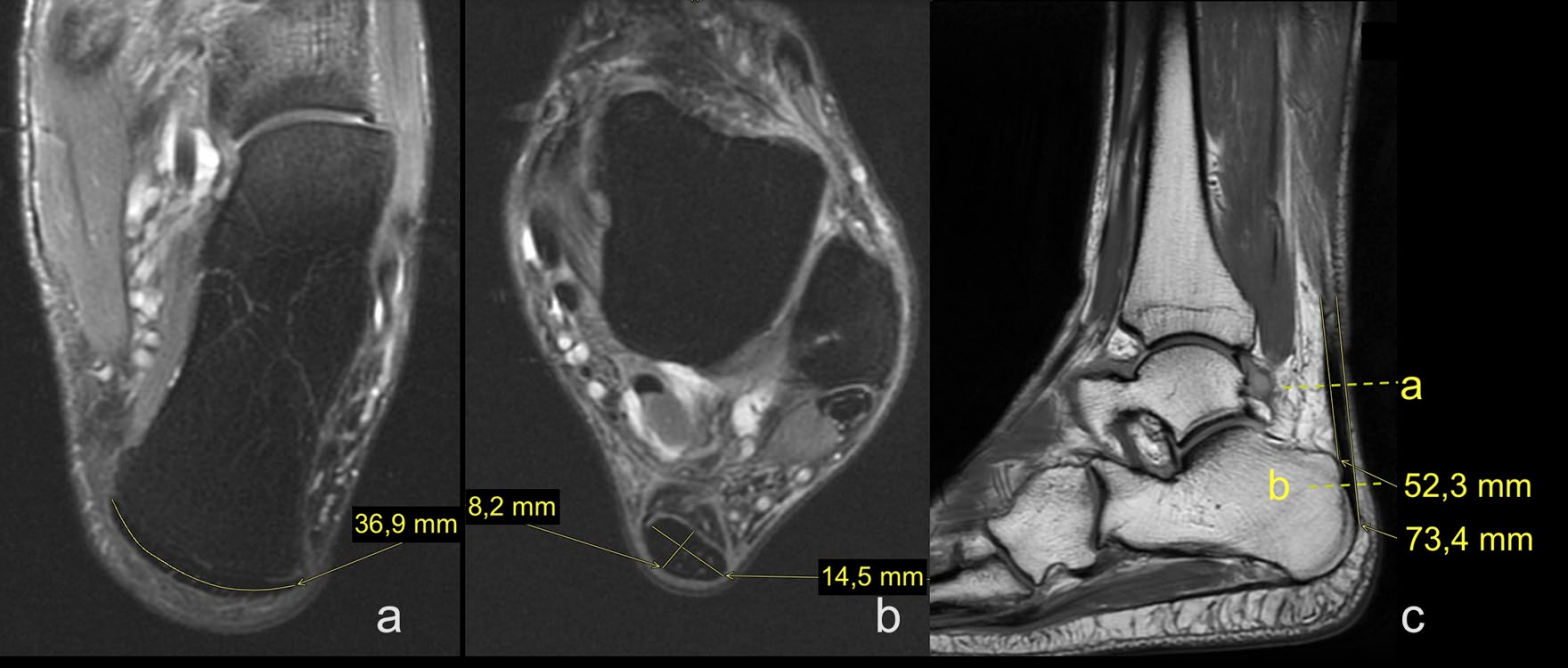
Diameters of the normal Achilles tendons in two patients. (**a**) and (**b**) a 34-year-old female with clinical suspicion of enthesopathy of the Achilles tendon, (**c**) a 28-year-old male with clinical suspicion of myositis. (**a**) and (**b**) axial section, PD-weighted FS, (**c**) T1-weighted.

**Figure 7:**
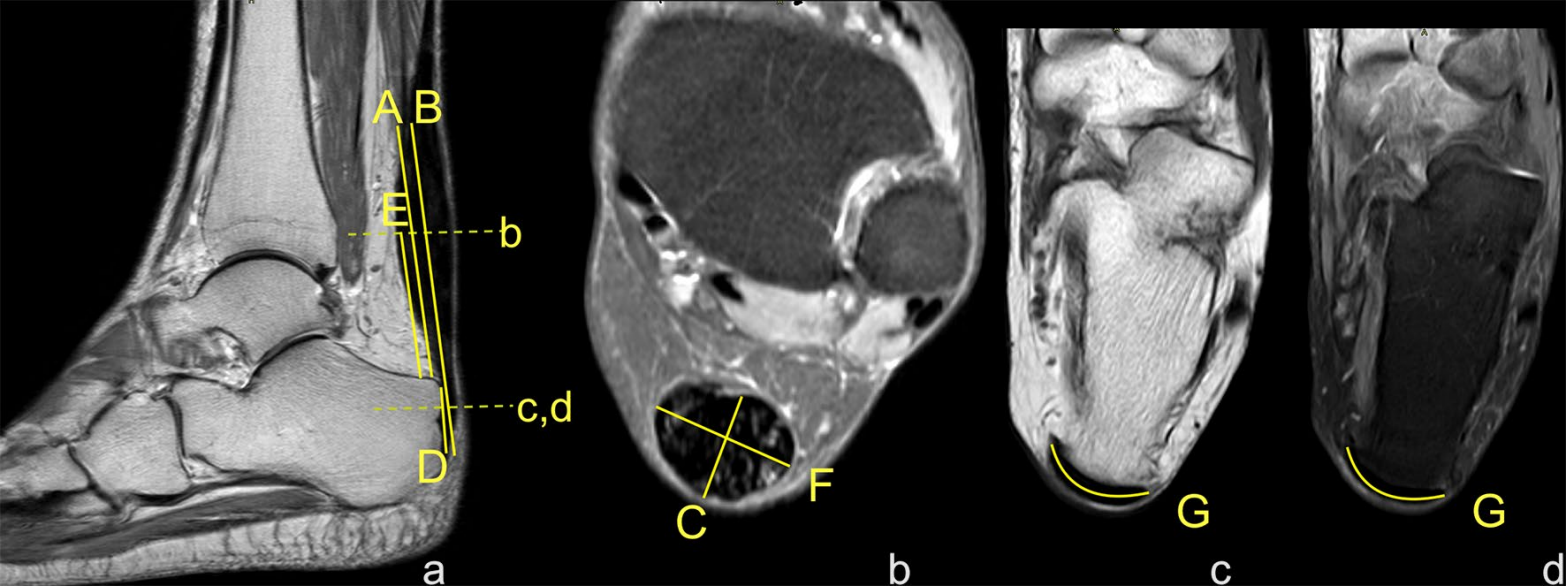
Diameters of the Achilles tendon with midportion tendinopathy of the left Achilles tendon in a 52-year-old male who presented with Achilles pain. Clinical suspicion of tendinopathy with partial rupture. MRI showed an advanced tendinopathy. A, B, C, D, F, G—dimensions of the Achilles tendon; definitions in the Material and Methods. (**a**) sagittal section PD- weighted, (**b**) PD- axial section weighted FS, (**c**) axial section T1- weighted, (**d**) axial section PD- weighted FS.

The highest ICC in the experimental group was noticed in the Achilles tendon length, 0.96 (0.89–0.98, SEM 2.22) for distance A and 0.95 (0.87–0.98, SEM 2.27) for distance B, Table 1. The lowest ICC in the experimental group was noticed in distance F at 0.72 (0.35–0.88, SEM 0.38), Table 1. In the control group the highest ICC was 0.96 (0.88–0.98, SEM 2.71) in distance A, while the lowest was 0.77 (0.53–0.90, SEM 0.4) in the distance F. The ICC and SEM for the experimental and control groups are presented in Table 1. The average thickness of the Achilles tendon midportion (diameter C) in tendinopathy was 11.2 ± 3.3 mm while in the control group this was 4.9 ± 0.5 mm, *p* < .001 (Tables 2, 3 and 4, Figs. 2 and 3). The average distance from the superior contour of the insertion to the level at which we assessed the thickness of the tendon (diameter E) was 24 ± 4.0 mm in the experimental group and 19.8 ± 2.2 mm in the control group, *p* < .001, Tables 2, 3 and 4. The average width of the midportion (diameter F) and insertion (diameter G) were larger in the experimental group, 17.2 ± 3.1 mm vs. 14.7 ± 1.8 mm for midportion, and 31.0 ± 3.9 mm vs. 25.7 ± 3.0 mm for insertion, respectively, *p* < .001, Fig. 4 and 5. The average length of the Achilles tendon was longer in patients with tendinopathy, i.e., diameter A: 60.0 ± 19 mm vs. 39.3 ± 14.1 mm, diameter B: 83.5 ± 19.3 mm vs. 61.5 ± 14.4 mm, *p* < .001 (Tables 2, 3 and 4, Figs. 2, 6, and 7).

**Table 1:** ICC and SEM of the normal tendon and tendinopathy changed the Achilles tendon.

Dimension	A	B	C	D	E	F	G
Tendinopathy	0.96 (0.89–0.98)	0.95 0.87–0.98	0.91 0.80–0.96	0.88 0.84–0.89	0.69 0.38–0.86	0.72 0.35–0.88	0.82 0.60–0.93
SEM	2.22	2.27	0.39	1.33	0.48	0.38	0.47

Dimension	A	B	C	D	E	F	G
Normal	0.95 0.88–0.98	0.93 0.84–0.97	0.89 0.75–0.95	0.82 0.46–0.93	0.88 0.71–0.95	0.77 0.53–0.90	0.86 0.69–0.94
SEM	2.71	2.69	0.12	1.26	0.98	0.40	0.84

A, B, C, D, E, F and G dimensions of the Achilles tendon defined in the Material and methods, ICC—interclass correlation coefficient, SEM—standard error of measurement.

**Table 2:** Dimensions of the Achilles tendon in the experimental and control groups.

	A	B	C	D	E	F	G
**Tendinopathy**							
Average	60.0	83.5	11.2	31.5	24.0	17.2	31.0
SD	19.0	19.3	3.3	6.1	4.0	3.1	3.9
Min	27.2	46.9	6.5	21.0	16.0	12.9	23.7
Max	106.8	132.6	21.2	45.0	32.4	26.3	42.1
**Normal**							
Average	39.3	61.5	4.9	18.3	19.8	14.7	25.7
SD	14.1	14.4	0.5	5.7	2.2	1.8	3.0
Min	8.5	28.8	3.9	4.5	14.6	11.2	15.2
Max	72.9	97.2	5.9	34.2	24.3	19.4	33.0
*p*	**	**	**	**	**	**	**

A, B, C, D, F, G- dimensions of the Achilles tendon; definitions in the Material and Methods. SD—standard deviation, min.—minimum, max.—maximum.

*p* < .001 marked as **.

**Table 3:** Dimensions of the Achilles tendon in the experimental group in subjects of different age groups.

Age		A	B	C	D	E	F	G
29–39	Average	57.1	79.2	11.0	32.8	23.0	17.3	29.6
	SD	17.2	15.8	2.9	7.4	4.0	4.1	2.8
	Min	33.2	51.2	8.2	21.3	18.4	12.9	25.8
	Max	79.3	98.8	15.9	43.0	29.1	24.7	33.5
40–49	Average	52.6	76.2	10.0	30.7	23.8	16.0	31.0
	SD	15.6	14.9	2.2	5.4	4.1	1.9	4.1
	Min	32.9	53.9	7.9	22.0	16.7	13.4	26.7
	Max	100.5	116.5	15.4	38.0	32.4	20.8	40.2
≥ 50	Average	63.2	86.9	11.6	31.7	24.3	17.6	31.2
	SD	19.5	20.4	3.6	5.9	4.0	3.2	3.9
	Min	27.2	46.9	6.5	21.0	16.0	13.2	23.7
	Max	106.8	132.6	21.2	45.0	32.1	26.3	42.1

A, B, C, D, F, G—dimensions of the Achilles tendon; definitions in the Material and methods, min.—minimum, max.—maximum, SD—standard deviation.

**Table 4:** Dimensions of the Achilles tendon in the control group in subjects of different age groups.

Age		A	B	C	D	E	F	G
18–29	Average	39.4	62.0	4.9	18.6	20.2	14.4	25.3
	SD	14.4	14.9	0.5	6.0	2.1	1.5	3.8
	Min	11.3	28.9	4.1	6.2	14.6	11.2	15.2
	Max	71.5	97.2	5.9	34.2	24.3	17.5	33
30–39	Average	41.6	63.8	5.0	17.9	20.0	14.5	26.6
	SD	14.2	14.5	0.6	5.5	2.2	2.0	2.2
	Min	20.6	42.1	3.9	8.5	15.3	11.3	22.5
	Max	66.3	89.6	5.8	26.5	23.5	18.2	29.5
40–49	Average	34.9	56.7	4.7	17.1	19.7	14.9	25.4
	SD	15.8	15.9	0.4	7.2	2.1	1.9	1.8
	Min	8.5	28.8	4.2	4.5	15.6	12.5	22.6
	Max	72.9	92	5.6	33.2	23.6	19.4	28.5
≥ 50	Average	41.6	63.4	5.2	19.5	18.7	15.5	26.1
	SD	12.8	13.4	0.3	4.2	1.7	1.6	2.4
	Min	17.7	42.2	4.6	11	16.2	12.3	20.7
	Max	61.5	86.4	5.5	25	21.6	18.2	29.4

A, B, C, D, F, G—dimensions of the Achilles tendon; definitions in the Material and methods, min.—minimum, max.—maximum, SD—standard deviation.

In the experimental group, the anterior tendon outline was most often convex n = 69 (93.2%) and less frequently flat n = 5 (6.8%). In the control group, we more often observed a concave shape n = 57 (70.4%), with a flat shape in n = 24 (29.6%).

We found no statistically significant differences in the dimensions of the Achilles tendon according to gender or side (*p* > .05).

We revealed the presence of correlations between the dimensions of the Achilles tendon in both the experimental and control groups; however, these correlations differed. Increased thickness of the midportion (diameter C) was related to the larger width of the midportion (diameter F). This tendency was significant in the experimental group (r = .49, *p* < .001) and almost non-existent in the experimental group (r = .01, *p* < .001), Table 5. The positive correlation between width (diameter G) and length of the insertion (diameter D) was noticed in tendinopathy but there was a negative correlation in normal tendons (r = .21 vs. r = − .23,* p* < .001). The width of the midportion (diameter F) and insertion (diameter G) was positively correlated with the length of the tendon (both with diameter A and B) in the experimental group, while in the control group, this correlation was negative, Table 5. Diameters A and B positively correlate with tendons thickness (diameter C) and the length of the insertion (diameter D) in both groups, Table 4. The correlation between the width of the midportion (diameter F) and insertion (diameter G) was more significant in the control group than in the experimental group, r = .57 vs. r = .28, respectively, *p* < .001, Table 5. We noticed a statistically significant negative correlation between the length of the insertion (diameter D) and its width (diameter G) in the control group and practically no such correlation in the experimental group (r = − .24 vs. r = .03, respectively, *p* < .001, Table 5).

**Table 5:** Pearson correlation coefficients between diameters of the Achilles tendon, *p* < .05 marked as *, *p* < .001 marked as **.

Tendinopathy N = 74	A	B	F	G
C	0.38	0.38	0.49	0.09
	**	**	**	**
D	0.63	0.61	0.21	0.03
	**	**	**	**
F	0.16	0.13	x	0.28
	**	**	x	**
G	− 0.07	− 0.05	0.28	x
	**	**	**	x

Normal N = 81	A	B	F	G
C	0.27	0.30	0.01	0.11
	**	**	**	**
D	0.85	0.83	− 0.23	− 0.24
	**	**	**	**
F	− 0.23	− 0.21	x	0.57
	**	**	x	**
G	− 0.18	− 0.16	0.57	x
	**	**	**	x

A, B, C, D, F, G—dimensions of the Achilles tendon defined in the Material and Methods.

## Discussion

To the best of our knowledge, this is the first MRI-based study regarding differences in Achilles tendon dimensions and correlations in normal tendons and tendinopathy. Our study revealed significant differences between the experimental and control groups in all studied dimensions correlations. Our results suggest that tendinopathy-related changes are not the simple enlargement of a healthy tendon in all dimensions but rather deformation occurring in each part differently. MRI was used before by many authors in assessing dimensions of the Achilles tendon because it is an appropriate and reliable method for dimensions evaluation^8^.

There are dimensions of the Achilles tendon that are related to its function. Previous results suggest that a longer Achilles tendon may help achieve superior running performance in endurance runners^16^. Increasing diameters are generally observed in degenerated tendons which exhibit reduced stiffness or ultimate tensile strength^17,18^.

Altered dimensions have an influence on correlations in normal and pathological tendons. The first difference in correlation was between the width of the midportion (diameter F) and the length (diameter A and B), which was positive in the experimental group and negative in the control group. The second difference in correlation was noted between the length of the insertion (diameter D) and the width of the midportion (diameter C) or insertion (diameter G), which was less significant in tendinopathy.

Diagnosis of early tendinopathy is challenging both clinically and in diagnostic imaging. When classical MRI sequences are used, a higher signal and thickening of more than 6 mm in the midportion of the Achilles tendon are hallmarks of tendinopathy^12^. At this stage, the patient usually reports pain in the Achilles tendon midportion, which is commonly related to sports activity. So far, attention has been focused on signal disturbances, which due to the short T2 time are relatively well visible in advanced tendinopathy. However, the early stage of tendinopathy is often silent both clinically and in MRI when classical sequences are used. Novel techniques such as T2 mapping and modifications of DTI MRI or spectroscopy MRI may help to identify subclinical tendinopathy; however, these are not used in clinical routine, mainly in research^19,20^.

We were unable to find any MRI-based studies on how tendinopathy's progression influences dimension correlations of the Achilles tendon. Based on a previous study, the thickness of the Achilles tendon insertion is correlated with body height^21^. There are also differences in the thickness of the Achilles tendon between populations. Based on ultrasound data, in the Chinese population, the average thickness of the Achilles tendon is 5.2 mm^22^, while in the European population it is 6.2 mm^12,23^. Most of the available studies regarding the dimensions of the Achilles tendon are based on ultrasound. However, values obtained by ultrasound cannot be directly applied to MRI. Most studies focus on the thickness of the Achilles midportion. Based on MRI studies, the average thickness of the midportion is 5.2 ± 0.77 mm^24^ or 6.0 mm^25^ in normal tendons and 7.6 ± 2.25 mm^26^ or 11.1 mm^25^ in symptomatic tendons. The selection of patients in previous studies was different, which makes comparison problematic. The average width of the Achilles tendon measured 3 cm superior to the calcaneus in an MRI study was estimated as 14.7 ± 2.06 mm in normal tendons^24^, which is similar to our results using a different method. Unfortunately, we were unable to find MRI studies assessing the width of the Achilles tendon insertion. Some authors use cross-section area (CSA) in morphological investigations of the Achilles tendon^8,27–30^. In Achilles tendinopathy, a more extensive cross-section was shown compared to normal tendons. Since CSA resembles an ellipse, this value depends linearly on the tendon anterior–posterior dimension and tendon width. We decided not to evaluate CSA in our study because our study focused on correlations between diameters. Moreover, in the literature, there are sufficient data concerning the value of the Achilles tendons CSA.

Ultrasound-based research of the normal Achilles midportion thickness are more available; the average thickness of the midportion is 4.3–4.7 mm^31,32^, while the width is 13.9 mm^33^ in athletes and 12–14.3 mm in the general population^31,34^. These values are similar to those obtained in our study. A tendency toward larger dimensions of the Achilles tendon in athletes was previously reported and was related to intensive training^35^. The dimensions of symptomatic tendons obtained by us are similar to a study performed by Neuhold et al.^25^, while they differ from some previous studies^26^. Differences in symptomatic tendons may result from different study inclusion criteria and population variability.

At present, 6 mm is considered as the normal thickness of the Achilles tendon^36^. Patients often present with a thickness over 8 mm, which corresponds to some irreversible alterations in tendon structure^36^. The Achilles tendon's thickness depends on age, physical activity, population factors, and anthropometric data^37^; hence there is a need for a population study on more extensive material that considers mentioned variables.

A spindle shape of the tendon influences the thickness value, so the level where it is measured is significant. As mentioned before, some authors use a constant level above the calcaneus in every examined case. The thickness part of the Achilles tendon differs among individuals. Thus, we evaluated diameter C on a non-constant level (diameter E). We believe that one constant measurement level can lead to an inaccurate assessment of tendon thickness.

We believe that measuring the thickness of a healthy tendon in the midportion should consider the variations of the soleus muscle^38^. Often, the distance of the lower outline of the soleus muscle to the calcaneus ranges from 2.54 to 7.62 cm based on anatomical study^38^. However, in 12.5% of cases, the distance between the soleus muscle and calcaneus is between 0 and 2.54 cm. The shortest distance seen in our study was identified in the control group and was 8.5 mm. The study cited above was performed on cadavers. The great advantage of anatomical research is the direct examination of muscles and tendons, but there is a risk of overdissection and problems related to shrinkage of tissues during the formalin fixation process.

The Achilles tendon insertion is a complex unit called an enthesis organ^39^. Due to the positioning of the ankle joint for MRI, our study measured the area where the Achilles tendon meets the calcaneus. It is difficult to distinguish the narrow layer of fibrocartilage from the closely adjacent part of the Achilles tendon. In practice, the measurement concerned the length of the enthesis organ, and not only the tendon-bone connection. The insertion length reveled in our study is slightly longer than previously reported by ultrasound or research performed on the cadavers^40^. The differences in methods and population differences may explain these discrepancies.

The other measurements reported in our study have not been reported previously in the available literature. This makes it difficult to fully discuss our results. It is known from previous studies that tendon thickness is influenced by several factors such as anthropometric factors, physical activity and limb dominance^22^. However, there are some studies, which have shown no significant differences in the thickness of the Achilles tendon between the dominant and non-dominant sides. In our study, we did not know which side was dominant. Differences in tendon thickness were not statistically significant between the right and left limbs.

Our study showed a positive correlation between the thickness of the normal Achilles tendon with its length, width of the midportion and insertion. We can therefore be suspected that other measurements may depend on height, training or domination. We see the need for further studies on a more homogeneous group. We included only patients with Achilles tendon thickness less than 6 mm^36^ in the control group. This was a criterion to avoid including patients with clinically and radiologically silent tendinopathy in the control group. We believe that, in some cases, before the signal change seen on conventional MRI techniques abnormality in proportions of the midportion and insertion may already be noticed. The normal Achilles tendon has a flat or concave anterior outline, while in tendinopathy a convex outline is usually seen^24^. We observed that, in tendinopathy, the anterior outline was usually convex, as reported in previous studies.

The Achilles tendon thickness assessment is clinically significant in predicting a clinical outcome. An MRI grading scale was developed by Nicholson et al.^35^ based on thickness of the Achilles tendon midportion and absence or presence of non-uniform degeneration^35,36^. Tendinopathy grading is related to prognosis for conservative treatment and is associated with clinical outcome^41^. In grade one, the dimension of the Achilles tendon varies between 6 and 8 mm and non-uniform degeneration is not visible, thus, conservative treatment is usually applied. The need for surgical treatment is higher in grades two and three. In grade two, the dimension of the Achilles tendon is more than 8 mm, and non-uniform degeneration constitutes less than 50%. In grade three, the diameter is more than 8 mm and non-uniform degeneration is more than 50%^36^. We could classify only two patients in our group as grade one tendinopathy. The aim of the diagnosis of early tendinopathy is to identify its presence at an early stage when no advanced degenerative remodeling of the tendon is present yet. Hopefully, it will be possible with novel MRI techniques in the future. Significant intratendinous aberrations and a thickness of more than 10 mm indicate the coexistence of tendinopathy and partial ruptures, which is irreversible in most cases ^9^. In our study, about 44% of the patients had an anterior posterior diameter of more than 10 mm, but no fluid signal, suggesting that a rupture was identified; thus, radiologic tendinopathy was diagnosed.

The differences in the dimensions between the groups with and without tendinopathy allow us to start a discussion on the inclusion of other dimensions than only the anteroposterior dimension in the criteria for diagnosing tendinopathy. The width of the midportion and insertion may be especially useful in cases when a higher tendon signal is not yet visible.

Considering the result of our study for tendon dimensions, we see the need for a prospective study involving patients with subclinical tendinopathy.

We realize that the indications for ultrasound and MRI of the Achilles tendon partly overlap. We decided to conduct our study based on MRI. The reason was because MRI is independent of the operator, unlike ultrasound. MRI is as effective as ultrasound in the detection of tendinopathy^9,10^. Interobserver variation in ultrasonographic measurements may be major even among experienced operators^42^. The disadvantage of MRI is the higher price and lower availability compared to ultrasound. Agreement between magnetic resonance and ultrasound in the tendon thickness evaluation has been estimated as excellent^43^. However, despite the many advantages of ultrasound and MRI, clinical examination is the basis for the routine diagnosis of tendinopathy^7^.

The potential clinical application of our results may diverge and can be used both by radiologists and other medical professions. We believe that the most important is the use in the early detection of Achilles tendinopathy. Our study highlighted the importance of the midportion width, indicating that a cross-section area that depends linearly on the tendon's width and thickness may be a valuable tendon condition indicator. The correlations we have demonstrated can provide valuable information in the monitoring of treatment response in tendinopathy. Due to the different correlations, we have demonstrated in both studied groups, it is  possible to identify subclinical tendinopathy cases in athletes. Another potential application of our results is for use in a biomechanical model of the normal and abnormal Achilles tendon.

We acknowledge several limitations in the present study. We used a routine Achilles tendon MRI protocol. The novel MRI sequences were not available that would eliminate patients with a subclinical phase of tendinopathy. It cannot be ruled out that the control group includes patients with subclinical tendinopathy. We have included the paratenon in our measurements. In the PD and T2-weighted images we used, it was challenging to distinguish the paratenon from the Achilles tendon. Some authors believe that T1-weighted images have a greater ability to differentiate between the paratenon and Achilles tendon^44^. In the protocol used here, T1-weighted images were most often taken only in the sagittal plane, which made it impossible to use them for measuring all dimensions of the tendon. It is difficult to estimate exactly how much the inclusion of paratenon influenced the dimensions of the Achilles tendon. According to previous studies, the average thickness of the paratenon in healthy people is 0.85 mm, and in tendinopathy it is about 1.33 mm^34^. Including the paratenon in calculating the cross-sectional area in the previous studies caused an overestimation of the value by about 20–40%^44^. The attachment measurement included the direct connection of the tendon with the bone and the area of the fibrocartilage layer on the calcaneus. Ultrasound could help distinguish between the part of the calcaneus covered by fibrocartilage and the connection between the tendon and bone^40^.

All examinations were performed as part of the clinical routine. The retrospective character and absence of surgical correlation are limitations of the study. We did not have sufficient information about the sports activity of the patients included in our study. All measurements also included the complex of the Achilles tendon and paratenon as it is difficult to distinguish a thickened tendon from the paratenon. Moreover, we did not know which limb was dominant. Further, no information regarding height and weight was available.

## Conclusion

The dimensions correlations in a normal Achilles tendon and tendinopathic tendon differ. The tendinopathy process is complex and represents not only a simple enlargement of the Achilles tendon, but changes the whole tendons' geometry as well. Further prospective studies on larger material regarding the tendon's dimensions should be performed, according to the on unified measurement protocol.

## Data Availability

The datasets generated during and/or analyzed during the current study are available from the corresponding author on reasonable request. Part of the cohort was used in a different study for a different purpose than in the current study; the manuscript is under consideration to be published in a different scientific journal.
